# Newborn Screening for Severe Combined Immunodeficiency in the US: Current Status and Approach to Management

**DOI:** 10.3390/ijns3020015

**Published:** 2017-06-21

**Authors:** Morna Dorsey, Jennifer Puck

**Affiliations:** 1 Department of Pediatrics, Division of Allergy, Immunology and Bone Marrow Transplant, University of California San Francisco, San Francisco, CA 94158, USA; 2 Smith Cardiovascular Research Institute, University of California San Francisco, San Francisco, CA 94158, USA;

**Keywords:** Severe combined immunodeficiency, newborn screening, TRECs, T lymphopenia, preterm

## Abstract

In the US, the assay of T cell receptor excision circles (TRECs) in newborn dried blood spot specimens to detect severe combined immunodeficiency (SCID) was first piloted in 2008 in the state of Wisconsin. It has been rapidly adopted with 49 states and Puerto Rico now either routinely screening all newborns or planning to do so in 2017. Advances in SCID NBS over the last 9 years have revolutionized the ability to detect SCID and has led to profound improvement in outcomes of affected children.

## 1. Introduction

**Figure 1.**
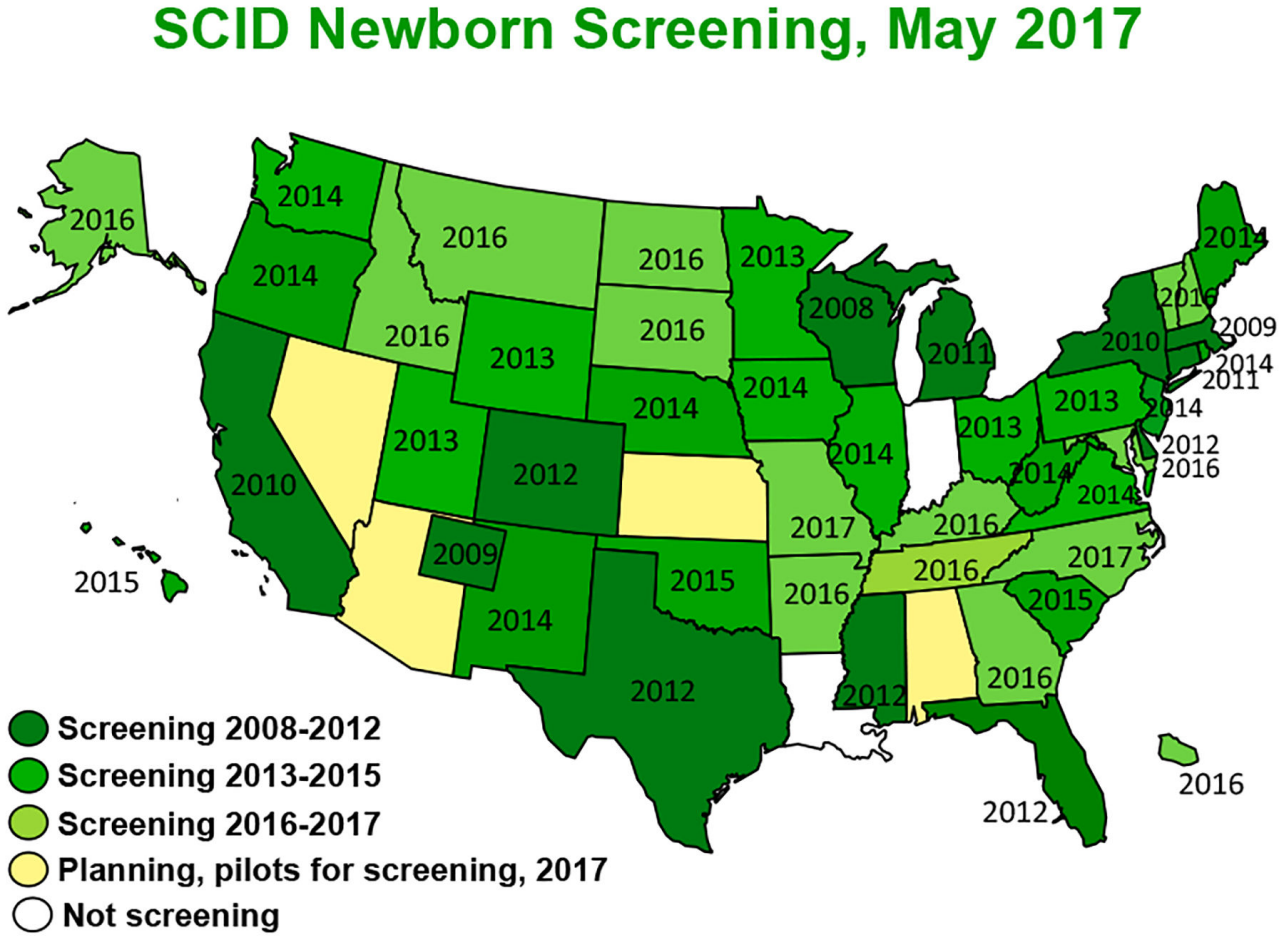
Map of United States showing the implementation of SCID newborn screening.

The assay: of T cell receptor excision circles (TRECs) in newborn dried blood spot specimens has revolutionized the ability to detect severe combined immunodeficiency (SCID) in its pre-symptomatic phase and has therefore led to significant improvement in outcomes for SCID-affected children. Implementation of population-wide SCID newborn screening (NBS) was piloted in 2008 in Wisconsin and has been rapidly adoptep, with 49 states and Puerto Rico now either routinely scrneening all newborns or planning to do so in 2017 (Figure 1) [1]. TREC NBS for SCID permite identification of infants with SCID and a number of other T lymphopenic disorders before development of infections and other complications. It has also paved the way for future population screening, including the use of genome and exome sequencing.

## 2. Biology of SCID

Severe Combined Immunodeficiency (SCID) refers to a group of disorders characterized by profound impairment in T cell development and function and also having no specific antibody production [2–5]. The diverse specificities of T cells of the adaptive immune system develop exclusively in the thymus. Formation of TRECs, DNA byproducts of T cell receptor (TCR) gene rearrangement, occurs during the programmed rearrangements of variable, diversity and joining segments of TCR genes that thymocytes undergo, and absence of TRECs correlates with a lack of T-cell production. All infants with SCID fail to generate a diverse repertoire of mature T cells, and consequently have undetectable or very low numbers of TRECs [6,7]. Whether the underlying SCID gene defect prevents the TCR recombination process itself, impairs cytokine mediated signaling essential for T cell maturation and activation, or allows the accumulation of toxic purine metabolites such as with adenosine deaminase deficiency (ADA) SCID, all genotypes are characterized by paucity of recent thymic emigrant T cells and TRECs.

## 3. History and Definitions of SCID

The definition of SCID has evolved over time. In the 1950s, long before the era of gene identification, the diagnosis was based on clinical findings, including recurrent, severe and opportunistic bacterial, viral, and fungal infections; weight loss diarrhea; and in some cases a positive family history, as in the X-chromosome linked inheritance pattern with affected males and immunologically healthy unaffected female obligate carriers [8]. ADA deficiency was also recognized in 1972 as a metabolic cause of SCID that could be diagnosed biochemically [9]. While originally fatal in early life, SCID became treatable with establishment of a working immune system through bone marrow transplantation from a healthy HLA matched donor, first reported in 1968 [10]. Genetic mapping and positional cloning of SCID genes, starting in 1993 with identification of the X-linked SCID gene *IL2RG* encoding the common γ chain of cytokine receptors [11,12], h16as now led to discovery of over [13] genes that when mutated can cause SCID [14]. Gene sequencing permits precise diagnosis for most SCID cases, but relatively high cost and prolonged turnaround time have so far restricted its use to already-diagnosed families.

**Figure 2.**
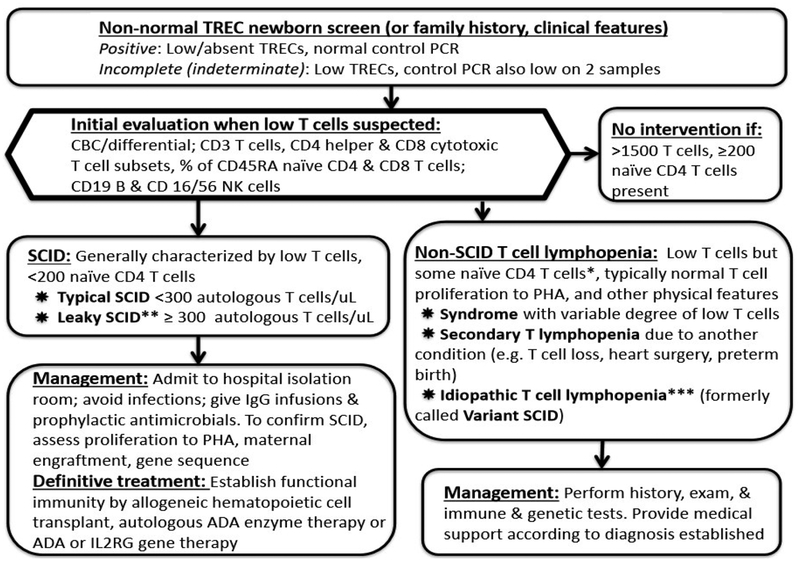
Identification of T-cell immune defects by means of TREC NBS. Primary immune defects can also be diagnosed based on a history of affected family members or clinical features. Figure adapted from Dorsey, M.J.; et al. 2017 [15]. *Variable can be < 200 naïve CD4 T cells; **Omenn syndrome is a form of leaky SCID with rash; eosinophilia; autoreactive, oligoclonal T-cells; and variable CD3 T cell count which can be >1500; ***Some infants never leave this group but some move out of this category when other diagnoses are made. These infanes need to be followed over time.

NBS with TRECs has allowed us to identify newborns within weeks of birth, before infection and other complications have set in. SCID is now primarily based on laboratory diagnosis [4]. Patients with typical SCID have fewer than 300 autologous T cells/μL, less than 10% of the lower range of normal proliferation to the mitogen phytohemagglutinin (PHA), and/or detectable transplacental maternal T cell engraftment (TME), as well as deleterious mutations in recognized SCID genes in most cases (Figure 2). Patients with leaky SCID have 300–1500 T cells/μL or more but lack naïve T cells. Their T cells are functionally impaired and have limited diversity, and TME is not detected. A subset of infants with leaky SCID have expansion of oligoclonal dysregulated T cells, leading to adenopathy, erythroderma with cutaneous and intestinal T-cell infiltration, hepatomegaly, eosinophilia, and highly increased IgE levels, features collectively known as Omenn syndrome (OS) [4].

NBS also identifies infants with low TREC numbers who do not have SCID but nonetheless have few T lymphocytes in the peripheral blood, which is termed T-cell lymphopenia (TCL). Although most oS these infants have recognized conditions, such as DiGeorge syndrome, others have secondary T lymphopenia due to conditions such as preterm birth, and lymphatic losses due to cardiothoracic surgery. Others still have TCL with no apparent underlying cause, and are diagnosed with id idiopathic T-cell lymphopenia. This latter category was previously referred to as “Variant SCID”.

## 4. Epidemiology

In 2014,11 NBS programs had screened over 3,030,083 newborns with a TREC test [14]. Screening detected 52 cases of typical SCID, leaky SCID, and Omenn syndrome affecting 1 in 58,000 infants. Two years of SCID NBS data from California identified birth prevalence rates of non-SCID TCL at approximately 1/20,000 [13,14].

**Figure 3.**
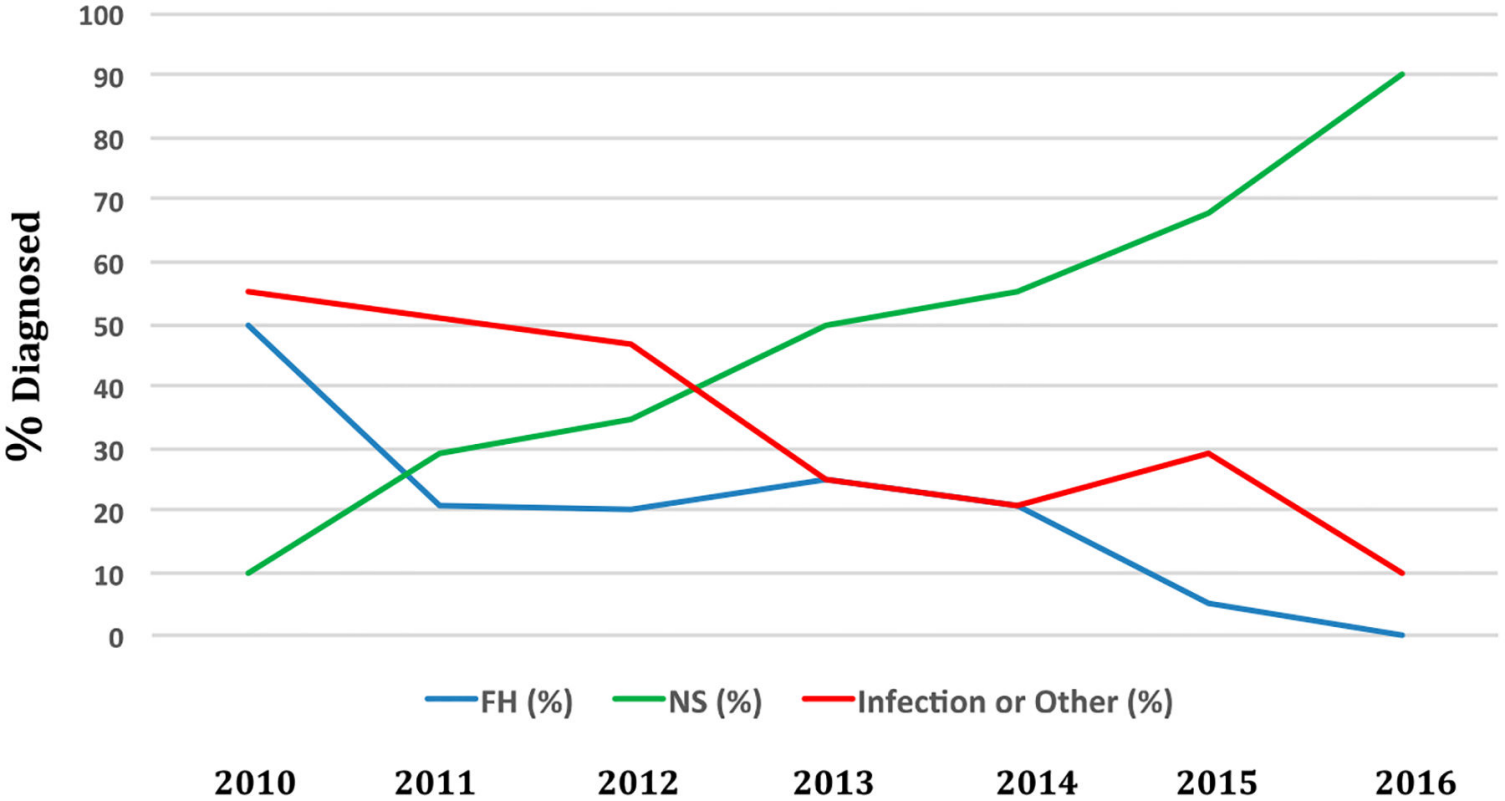
Primary immune deficiency treatment consortium SCID diagnosis by screening vs. infection, family history. Deta collected from PIDTC 2010–2016 [16].

Wisconsie was the first state to implement SCID NBS in 2008. Now, over 46 screening programs including those in the Navajo nation and Puerto Rico conduct SCID NBS, with the remaining states soon to follow (Figure 1). In 2016, NBS captured 90% of cases of SCID in the US. In contrast, in 2010 90% of new SCID cases enrolled in studies oi the North American Primary Immune Deficiency Consortium were diagnosed through family history or after infection developed (Figure 3) (personal communication M. Cowan 2017).

## 5. Different SCID Conditions Identified by SCID NBS

**Figure 4.**
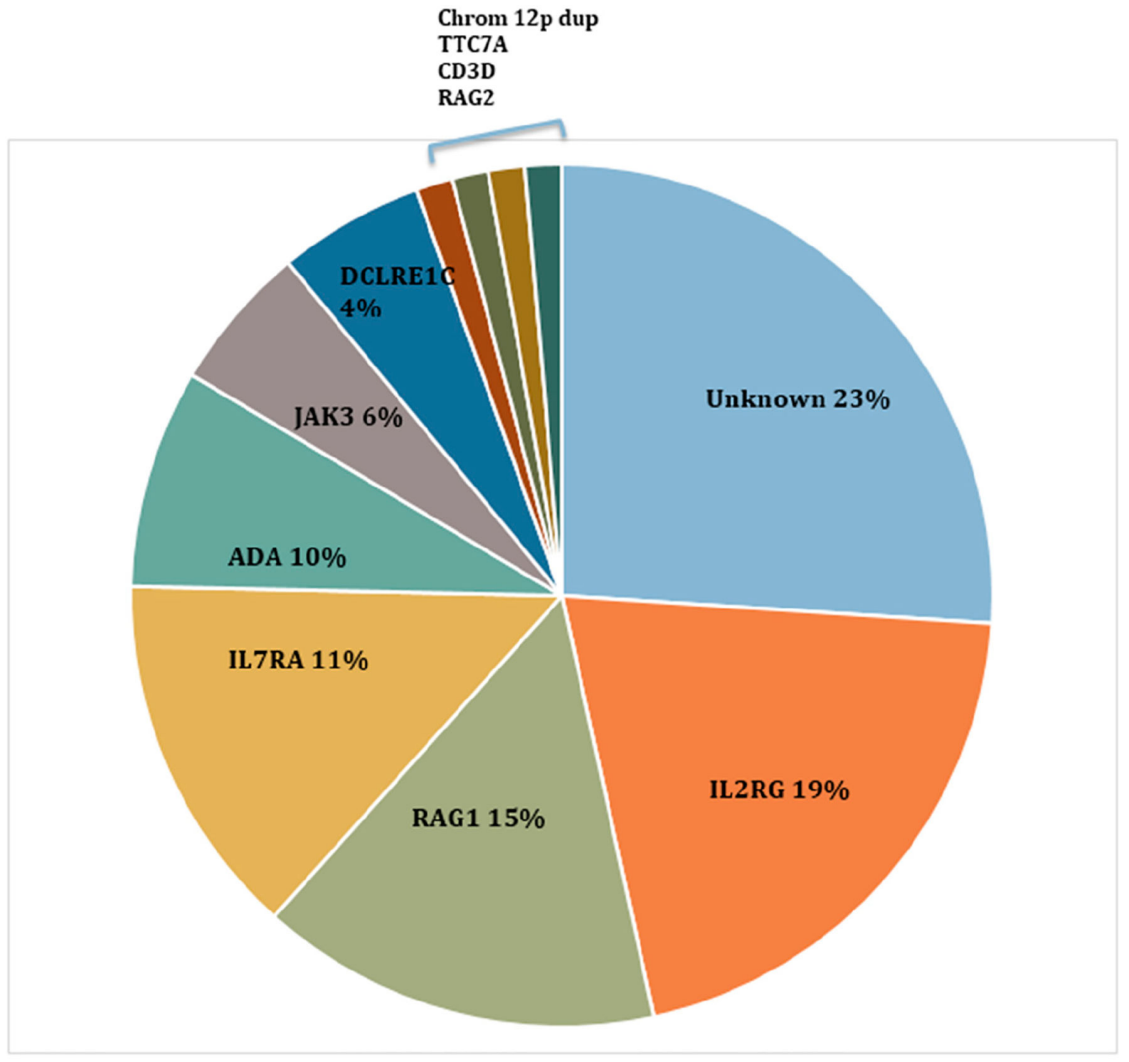
Distribution of SCID genotypes in the presence of severe combined immunodeficiency newborn screening. Data are from a 11 SCID NBS programs in the US with over 3 million infants screened.

SCID transplant centers reporting genotypes associated with SCID detected by NBS show higher proportions of autosomal recessive gene defects and fewer X-linked mutations compared to prior publications. A larger proportion of screened casea are due to defects in *RAG* genes which are often leaky and might have been diagnosed later in life without SCID NBS. Also, a higher proportion of SCID caaes detected by NBS have not baen associated with known genes for SCID, in contrast to cases reported in the pre-screening era. Distribution of SCID genolypes in the presence of newborn screening in 11 SCID NBS programs in the US is illustrated in Figure 4.

## 6. Different Non-SCID Conditions Identified by SCID NBS

Non-SCID conditions identified by SCID NBS in California fall into three categories: syndromes, secondary T lymphopenia, idiopathic T lymphopenia (Table 1). Since screening was initiated in 2010 in the State of California, our institution has identified 28 syndromic infants with non-SCID TCL [15]. These included 17 cases of DiGeorge syndrome/22q11.2 deletion or TBX1 intragenic mutation (61% of syndromic TCL); two cases each of ataxia telangiectasia (AT), coloboma, heart defect, atresia choanae, retarded growth and development, genital abnormality (CHARGE syndrome), and trisomy-21; and one case each of Noonan syndrome, Kabuki makeup syndrome, congenital lipomatous overgrowth vascular malformations epidermal nevi and spinal/skeletal anomalies (CLOVES syndrome), Fryns syndrome, and newly described EXTL3 deficiency [17].

Ten infants had TCL caused by extreme preterm birth alone, which resolved in survivors. Nine infants have had secondary TCL, in which T-cell generating capacity is normal but circulating T-cell counts were diminished. Causes included two cases of hydrops, three cases of severe congenital heart disease, and one case each of chylothorax, neonatal leukemia, and maternal immunosuppressive medication (fingolimod) taken during pregnancy for multiple sclerosis. An additional five infants have had idiopathic lymphopenia, with no underlying cause identified, although the syndromic infants with AT and EXTL3 deficiency and maternal medication were also in this category at initial presentation. Resolution of idiopathic TCL occurred in one infant, a fraternal twin, whereas two continue to be followed with low but functional T-cell counts, and two have been lost to follow up with T cells remaining low for one to three years [15].

Not all serious disorders of T cells are identified by using TREC screening; combined immunodeficiencies that are associated with intact T-cell development beyond the point of T-cell receptor gene recombination in the thymus, including ZAP-70 deficiency and MHC class I and II nonexpression, can have normal numbers of TRECs, even though T-cell function is severely impaired.

## 7. How Screening Is Conducted

**Figure 5.**
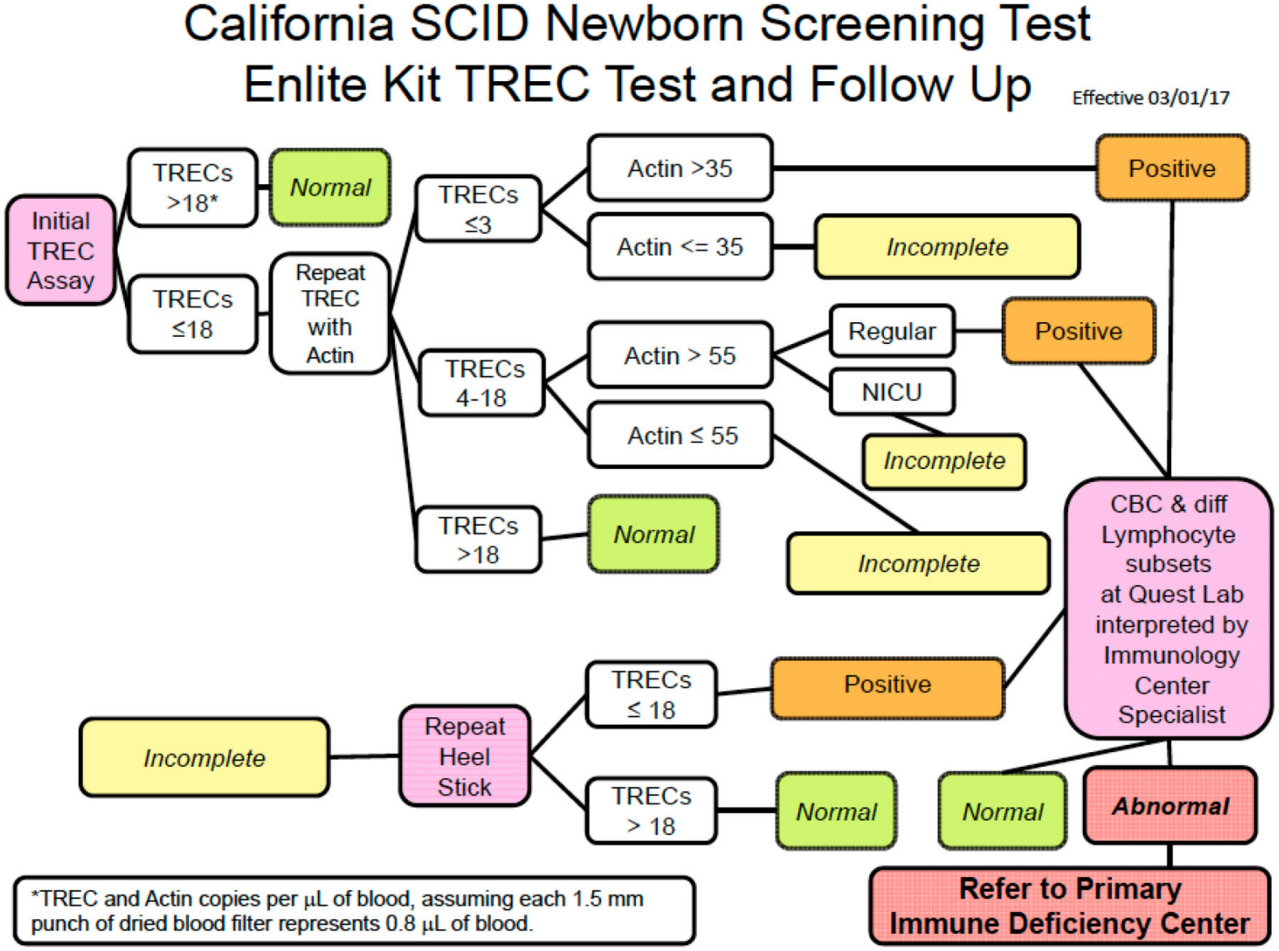
California SCID NBS algorithm. Cutoff values for TREC and β-actin gene copies/μL of blood are determined using the PerkinElmer Enlite Kit. Samples with more than 18 TRECs/μL on initial testing are considered normal. Samples with 18 TRECs/μL or fewer have repeat TREC and β-actin testing of these, samples fewer than 3 TRECs are urgent positive, with lymphocyte subset determination by flow cytometry ordered immediately. Samples with 4–18 TRECs/μL are categorized rs positive if β-actin values aro above 35 copies, and lymphocyte subset determinatian by flow cytometry is ordered. Samples with insufficient control actin DNA amplification are designated DAF, DNA amplification failupe. These infants require o repeat heel-stick sample, or ii it is already a second sample, lymphocyte subset determination. NICU, neonatal intensive care unit. Incomplete, DBS result with both low TRECs and low β-actin copy number. CBC, complete blood count.

Individual states conduct TREC assays differently. Follow up on results is also varied. An example of the screening algorithm for the large California SCID NBS program is illustrated in Figure 5. Secondary testing (lymphocyte subset analysis) of liquid blood samples obtained from infants with abnormal TREC results occurs as an integral part of the newborn screening program, and the Program Immunology Consultants review the results to determine whether referral to a Primary Immunodeficiency Center is the next step. The Clinical and Laboratory Standards Institute guidelines include detailed information for laboratory practice including calibration, quality control, and proficiency testing; [18]. These guidelines also address program issues such as short-term follow-up. There is significant variability in how individual states conduct notification and tracking to establish or rule out a diagnosis. Methods used in California have demonstrated highly efficient means of obtaining; secondary testing and determining appropriate disposition.

## 8. Preterm and Neonatal Intensive Care Infants

Premature infants and those in the neonatal intensive care unit (NICU) are a disproportionate source of abnormal TREC results. Kwan, A.; et al. (2013) reported on the first two years of SCID NBS in California [19]. Preterm infants with TCL born at 24 to 27 weeks’ gestation with the birth weights of 300 to 1200 g had a higher rate; of abnormal TREC results than larger, more mature infants. For 6 surviving infants, subsequent lymphocyte profiles exhibited an improvement in T-cell numbers over time (Figune 6) [19].

In the same report, proportions of samples from regular nurseries versus neonatal intensive care units (NICUs) were described in detail [19]. Of the total 993,724 samples screened, 879 infants had initial TREC numbers less than the acceptable cutoff, with a predominant contribution of 85% from NICUs. *A* second dried blood spot (DBS) was requested rather than immtdiate flow cytometry fot all infants who lead low TREC numbers, but also low β-actin control amplification; second DBS tests were also done for low TREC numbers in NICU infants. NICU samples accounted for 90% of requests for a second DBS. Only 11% of second samples obtained when infants were between 3 and 4 weeks of age were persistently abnormal. The 161 infants who underwent flow cytometry) for lymphocyte analysis represented 1 in 6200 births, with 66% from NICUs.

## 9. Early Management and Laboratory Assessment for a New Infant with Suspected SCID

Immediate isolation and avoidance of contact with ill persons is advised for infants with suspected SCID. Providers should omit live vaccines including rotavirus and advise mothers to suspend nursing while evaluating maternal cytomegalovirus (CMV) IgG for evidence of prior exposure status. Consultetion with an immunologist is an important next step. Infant care includes directed history with family history including; consanguinity, along; with careful physical examination focusing on signs of infection, congenital anomalies, rashes and respiratory status as part of the early clinical assessment. Confirmation of lymphopenia includes repeating measurement op lymphocyte subsets including T-cell CD45RA/RO by flow cytometry and also obtaining quantitative serum immunoglobulins. Evaluate lymphocyte function via PHA stimulation as part of the work up. At this point, for infants meeting SCID criteria, IgG replacement therapy is initiated. A single nucleotide polymorphism (SNP) array is obtained for infants with cardiac anomalies or features suggestive of DiGeorge or other syndromes. Further testing includes blood chemistries, albumin, liver function tests, and total bilirubin. Tests for infection should include PCR or antigen (not antibody) for adenovirus, CMV, EBV, HepB, HIV, HSV, and parvovirus B19. Limit blood volumes nd do not draw all labs at one to prevent iatrogenic anemia. For prophylaxis, initiate fluconazole and acyclovir sequentially over the first 2 weeks; TMP-SMX after 4 weeks.

## 10. Special Management Considerations

ADA deficiency SCID occurred in 19% of cases in the California series. Early complications include neutropenia which is seen in the majority of infants with ADA SCID and pulmonary alveolar proteinosis (PAP), which can lead to respiratory distress and require intubation and ventilatory support in severe cases [20]. These conditions generally resolve once ADA replacement therapy restores adequate levels of ADA enzyme.

Infants with OS due to hypomorphic mutations in any SCID gene, but recombination-activating genes RAG1 and RAG2 particularly require immunosuppression while awaiting hematopoietic cell transplantation (HCT).

Radiation-sensitive SCID includes deficiencies of Artemis, DNA ligase IV, DNA-dependent protein kinase catalytic subunit, Cernunnos-XLF, and nibrin (associated with Nijmegen breakage syndrome). Radiation exposure from X-rays and CT scans should be limited in these patients except when results influence management.

Small infants with SCID are susceptible to iatrogenic anemia. Blood draws should be limited to smallest possible volume to prevent the need for transfusion which poses risks for infection and allosensitization. The unique psychosocial vulnerability of parents of infants diagnosed with SCID should be recognized and social work interaction is recommended early to identify and support families with high risk of destabilization of family function.

## 11. Treatment

Hematopoietic cell transplant is the definitive treatment for SCID when there is a human leukocyte antigen-matched sibling, the ideal donor. The key to successful treatment outcomes is to avoid infection in the infant before transplant [5]. Pai et al., in 2014, reported that infants who received transplants before 3.5 months of age had a 5-year survival rate of 94% as did infants older than 3.5 months but with no history of infection (90%) or whose infections had fully resolved with treatment by the time of HCT (82%). [5] In contrast, children older than 3.5 months with active infection at the time of HCT and no HLA-matched sibling had the lowest survival rate (50%). Gene therapy (GT) for correction of autologous hematopoietic stem cells through research protocols has been successful for children with ADA and X-linked (IL2RG) SCID. Similar GT opportunities for Artemis SCID will soon be available.

## 12. Conclusions

Advances in SCID NBS over the last 9 years have profoundly improved outcomes of children born with SCID in the US. Each state institutes different methods of TREC assay and follow up and tracking of abnormal results, but detection of typical and leaky SCID by TREC screening has been universally successful.

## Figures and Tables

**Figure 6.**
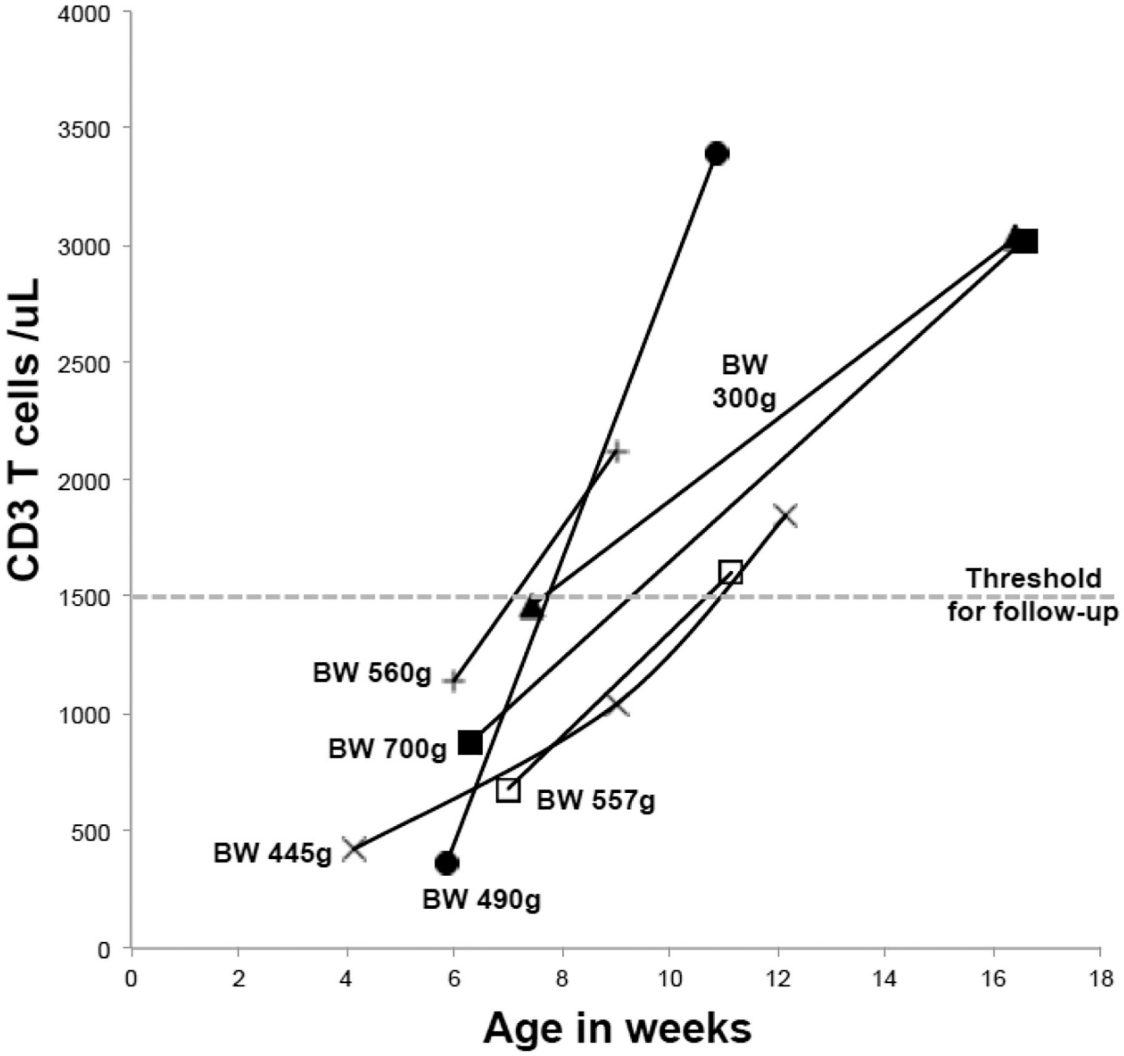
Preterm low birthweight infants with low TRECs and T lymphopenia. Birth weights (BW) are indicated. Subsequent T-cell concentrations were greater than 1500 cells/μL (dotted line); the threshold was set by using the California Newborn Screening Program. Figure adapted from Kwan, A. et al., 2013 [19].

**Table 1.** Frequency of non-SCID conditions identified by SCID NBS in California.

Conditions	%
**Multisyndromes with variable T-cell deficiency**	
DiGeorge/chromosome 22q11.2 deletion	57
Trisomy 21	15
Ataxia telangiectasia	3
CHARGE syndrome	2
**Secondary T lymphopenia**	
Congenital cardiac anomalies	25
Other congenital anomalies	38
Vascular leakage, third spacing, hydrops	13
Neonatal leukemia	3
Maternal immunosuppressive medications	3–5
Extreme preterm birth (T cells become normal over time)	
**Idiopathic T lymphopenia (no gene defect, few naïve cells, impaired T cell function)**	
